# Clay versus Gravel

**Published:** 1902-02-22

**Authors:** 


### The Hospital

A Journal of

### The Medical Sciences and Hospital Administration.

Vol. XXXI.—No. 804.] Edited by Sir Henry Burdett, K.C.B., and Solomon C. Smith, M.D., M.R.C.P. [February 22, 1902.

### CLAY VERSUS GRAVEL.

The Balneological and Climatological Society has lately been discussing a question of much interest to all who are in search of a house in London, namely, whether it is best to live on clay or upon gravel. In the opinion of the public, so far as we can estimate that opinion by the advertisements of the wily house builder, the question has long since been decided in favour of gravel. Whoever saw an advertisement giving, among the other advantages of a residence, that it was built upon a stiff clay soil? Gravel is what the public asks for, gravel is forced upon us by every house agent, and it is to the pile of gravel which he tells us he has dug out of our foundations that the builder points as the final assurance that if we do but buy his trumpery villa we shall have health and happiness ever after. All of which, if it do not prove the honesty of the builder, at least shows the public confidence in gravel. Dr. Clippingdale, however, has satisfied himself that, "in estimating the relative advantages of dwelling in London on clay or on gravel soil, the statistics are in favour not of gravel but of clay." Before, however, we accept such a conclusion it may be as well to note that the statistics referred to are of an extremely simple character, and that the manner in which they are applied to the problem is elementary, to say the least of it. What Dr. Clippingdale did was to compile a list of the sanitary areas of London, giving their soil, elevation, population and death rate, and arrange them in the order of their death rate. The result is certainly sufficiently striking. Beginning with Hampstead, the soil of which he describes as "clay, generally capped in places by Bagshot sand," and the death rate of which is given as 11·3, he descends through the various parishes, and ends with St. Luke, the soil of which is gravel and the death rate 28·8, and it must in truth be admitted that clay does figure most conspicuously among the districts with the low death rates, and that gravel is infinitely the most common form of soil among those in which the death rate is the highest. In fact, work it out every way we will, gravel comes out very poorly. Whether we take the general death rate or that from zymotic diseases, or from phthisis, or from other respiratory diseases; in each case the highest rates are on the gravel or alluvium, and the lowest upon clay, so far, that is to say, as Hampstead may be regarded as clay. A very slight consideration of the problem, however, is sufficient to show that other and far more important factors than condition of soil are engaged in the determination of the varying death rates which prevail in different parts of London. Elevation above the river level, density of population, the condition of the inhabitants in regard to wealth or poverty, the nature of their work, the character of their dwellings, and the degree of overcrowding which prevails, are all factors of great importance, and as in London these factors intrude at every point, they very seriously whittle away the importance of any broad generalisation derived from the coincidence of high death rates with gravel soil. In fact, Dr. Clippingdale himself concludes that in estimating the salubrity of any district it is more important to attend to elevation than to soil. As to any attempt to apply to other districts conclusions arrived at regarding London, it must be remembered that much of the gravel in London is gravel at its worst. The central plateau, which stands from 50 to 80 feet above the sea level, has for long been covered with houses, and the subsoil has become fouled by centuries of cesspools, while in the out districts, most of what is called gravel is but a thin layer, partly brick earth and partly alluvium, the water in which is held up by the underlying clay, a form of soil which has never been regarded as healthy, wherever it occurs.

On the other hand, clay as met with in London is often clay at its best. The evil reputation of clay has arisen from experience of extensive, often flat and ill-drained districts, on which the water lodges, and the soil of which remains damp for weeks after a good wetting. But most of the clay areas in London are on the slope. Moreover, most of them, as Hampstead, Highgate, Sydenham, Notting Hill, etc., are fairly elevated, much more so than the low-lying gravels; while in proportion as the London area has become paved and built over, the special evil of clay, that is its prolonged wetness after rain, only shows itself in a very modified degree; so that we certainly must not apply to other districts anything that statistics may seem to teach as to the comparative healthiness of clay in London. The truth probably is, that if all London were properly built—the houses with air-tight foundations and the drains with water-tight joints—the subsoil would be a matter of comparative indifference. Unfortunately the houses are not well built, nor are the drains properly laid, and under such conditions the dweller upon porous gravel has to suffer from his neighbours' sanitary sins as well as his own, while the dweller on stiff clay, however bad his neighbours' drains may be, obtains some temporary protection by the impervious nature of the soil on which his foundations rest. This probably is the explanation of such of the London mortality as may properly be laid at the door of gravel soil.

